# *Perilla frutescens* Leaf Extract and Fractions: Polyphenol Composition, Antioxidant, Enzymes (α-Glucosidase, Acetylcholinesterase, and Tyrosinase) Inhibitory, Anticancer, and Antidiabetic Activities

**DOI:** 10.3390/foods10020315

**Published:** 2021-02-03

**Authors:** Zhenxing Wang, Zongcai Tu, Xing Xie, Hao Cui, Kin Weng Kong, Lu Zhang

**Affiliations:** 1 College of Chemistry and Chemical Engineering, Jiangxi Normal University, Nanchang 330022, China; wangzhenxingfood@163.com (Z.W.); zhanglu00104@163.com (L.Z.); 2 College of Life Sciences, Southwest Forestry University, Kunming 650224, China; 3 College of Life Sciences, Jiangxi Normal University, Nanchang 330022, China; cuihaoeric@jxnu.edu.cn; 4 National R&D Center for Freshwater Fish Processing, Jiangxi Normal University, Nanchang 330022, China; 5 State Key Laboratory of Food Science and Technology, Nanchang University, Nanchang 330047, China; 13609092537@126.com; 6 Department of Molecular Medicine, Faculty of Medicine, University of Malaya, Kuala Lumpur 50603, Malaysia; kongkm@um.edu.my

**Keywords:** *P. frutescens* leaf, phytochemical composition, antioxidant activity, enzyme inhibitory, anticancer ability, antidiabetic activity

## Abstract

This study aims to evaluate the bioactive components, in vitro bioactivities, and in vivo hypoglycemic effect of *P. frutescens* leaf, which is a traditional medicine-food homology plant. *P. frutescens* methanol crude extract and its fractions (petroleum ether, chloroform, ethyl acetate, n-butanol fractions, and aqueous phase residue) were prepared by ultrasound-enzyme assisted extraction and liquid–liquid extraction. Among the samples, the ethyl acetate fraction possessed the high total phenolic (440.48 μg GAE/mg DE) and flavonoid content (455.22 μg RE/mg DE), the best antioxidant activity (the DPPH radical, ABTS radical, and superoxide anion scavenging activity, and ferric reducing antioxidant power were 1.71, 1.14, 2.40, 1.29, and 2.4 times higher than that of control Vc, respectively), the most powerful α-glucosidase inhibitory ability with the IC_50_ value of 190.03 μg/mL which was 2.2-folds higher than control acarbose, the strongest proliferative inhibitory ability against MCF-7 and HepG2 cell with the IC_50_ values of 37.92 and 13.43 μg/mL, which were considerable with control cisplatin, as well as certain inhibition abilities on acetylcholinesterase and tyrosinase. HPLC analysis showed that the luteolin, rosmarinic acid, rutin, and catechin were the dominant components of the ethyl acetate fraction. Animal experiments further demonstrated that the ethyl acetate fraction could significantly decrease the serum glucose level, food, and water intake of streptozotocin-induced diabetic SD rats, increase the body weight, modulate their serum levels of TC, TG, HDL-C, and LDL-C, improve the histopathology and glycogen accumulation in liver and intestinal tissue. Taken together, *P. frutescens* leaf exhibits excellent hypoglycemic activity in vitro and in vivo, and could be exploited as a source of natural antidiabetic agent.

## 1. Introduction

Type 2 diabetes mellitus (T2DM), which is characterized by postprandial hyperglycemia and chronic hyperglycemia, is one of the most serious metabolic diseases worldwide and its pathogenesis is associated with oxidative stress [1]. Oxidative stress is an imbalance between reactive oxygen species (ROS) and cellular antioxidative systems; excessive ROS accumulation induces oxidative stress, leading to cell damage, which in turn is involved in diabetes and various complications [2]. Furthermore, many other metabolic diseases are also associated with oxidative stress, including obesity, cancer, neurodegenerative disorders, and cardiovascular complications [3]. Indeed, these diseases also influence each other through the metabolic abnormalities associated with oxidative stress, for example, there is a close and direct link between diabetes and Alzheimer’s disease (AD) [4]. In addition, cancer development may lead to the aggravation of an underlying diabetic condition [5]. Consumption of antioxidant-rich foods has been considered to have the ability to potentially modulate oxidative stress, which in turn, has beneficial impacts on oxidative stress-related diseases [6]. Natural antioxidant products, especially polyphenol (flavonoid)-rich sources, such as edible medicinal herbs, fruits, vegetables, cereals, teas, and seeds, have attracted more attention because of their advantages in safety, effectiveness, and non-toxic properties. Ever-growing consumer demand for healthy, combined with a preference for natural ingredients, has led to very big growth in plant-based extracts functional foods and dietary supplements markets in recent years, especially during coronavirus disease 2019 (COVID-19) outbreak [7,8,9,10].

*P. frutescens* (L.) Britt. is a common annual Labiatae planted in the Southeast Asian region, and the most frequently used part is the leaf. In addition to the use as a green leafy vegetable, spice, and flavoring agent in food processing, *P. frutescens* leaves are widely used in traditional Chinese medicine to treat common cold, diarrhea, fever, cough, vomiting, and more. Modern pharmacological studies have demonstrated that *P. frutescens* leaves contain rich bioactive components, like phenolics, flavonoids, anthocyanins, tannins, and essential oil, and exhibited a variety of activities, including antioxidant, antiallergy, antiinflammation, antitumor, and antibacterial activities [11,12,13,14]. In our previous study, the methanol crude extract of *P. frutescens* leaves exhibited a promising α-glucosidase inhibitory ability after an in vitro gastrointestinal digestion, which exhibited the therapeutic potential in diabetes [15], this was also evidenced by other previous research [16,17]. However, the majority of studies on the hypoglycemic effect of *P. frutescens* leaves have been conducted in vitro, with very little report conducted at the animal level.

In this study, the antioxidant activities, α-glucosidase, acetylcholinesterase, tyrosinase inhibition abilities, and anticancer abilities of *P. frutescens* leaves extracts prepared with solvents at different polarities were determined in order to assess their abilities in preventing and treating oxidative stress-related diseases. Then, the fraction with high content of phenolics and flavonoids and good bioactivity were quantified by high-performance liquid chromatography (HPLC), and its antidiabetic effects were further evaluated using diabetic rats. The findings of present work could provide a new reference for the development of novel antidiabetic drugs.

## 2. Materials and Methods

### 2.1. Chemicals and Reagents

All chemical reagents including methanol, petroleum ether (PE), chloroform (CF), ethyl acetate (EtOAc), and n-butanol (n-BuOH) were purchased from Sinopharm Chemical Reagent Co., Ltd. (Shanghai, China). Folin-Ciocalteu, pyrogallol, and other analytical grade chemicals were purchased from Aladdin (Shanghai, China). Chromatographic acetonitrile was purchased from Merck (Darmstadt, Germany). Gallic acid and other standards for HPLC were purchased from Yuanye Bio-Technology (Shanghai, China). Cellulase (3000 U/g) and pectinase (40,000 U/g) were purchased from Xiya reagent (Shandong, China). α-glucosidase from Saccharomyces cerevisiae (G5003), acarbose, 4-methylumbelliferyl α-D-glucopyranoside (4-MUG), acetylcholinesterase (AChE) from Electrophorus electricus (C3389), galanthamine (GAL), acetylthiocholine iodide (ATCI), 5,5-dithiobis-(2-nitrobenzoic acid) (DTNB), tyrosinase from mushroom (T3824), kojic acid, 3,4-Dihydroxy-L-phenylalanine (L-dopa), cisplatin, and streptozocin (STZ) were purchased from Sigma-Aldrich (St. Louis, MO, USA).

The MCF-7 and HepG2 cells were purchased from Conservation genetics CAS Kunming cell bank. Dulbecco’s modified Eagle’s medium (DMEM), fetal bovine serum (FBS), and other cell culture reagents were purchased from Gibco (Carlsbad, CA, USA). Cell counting kit-8 (CCK-8) reagent was purchased from Dojindo (Kumamoto, Japan).

### 2.2. Samples Preparation

The leaves of *P. frutescens* (L.) Britt were purchased from a farm (24°32′44″ N, 116°15′12″ E) in Meizhou city, Guangdong, China. Extraction was done by referring to the previous method [18]. Briefly, 1.5 kg of sun-dried powdered leaves (moisture content ranges of 10–14%) was mixed in a 6-fold volume (volume to mass ratio) of pure water, the solution was adjusted to pH of 5 with hydrochloric acid (HCl 1 M), then 0.67% cellulase and 0.05% pectinase were added, followed by ultrasound-assisted enzymatic hydrolysis was performed at 400 W, 50 °C for 60 min. After hydrolysis, methanol was added until 70% final concentration and the solution volume was adjusted to 20-fold volume (70% methanol volume to samples mass ratio). Subsequently, ultrasound-assisted extraction of this mixture was performed two times in the same condition as before. The extract was evaporated under reduced pressure at 50 °C to yield 198 g of dry crude extract (CE), which was then suspended in 5 L H_2_O and extracted twice with an equal volume of organic solvent of different polarities successively, and the solvents used for the extractions were PE (polarity 0.1), CF (polarity 4.1), EtOAc (polarity 4.4), and n-BuOH (polarity 3.7). The resulting solvent fractions were collected and all solvents were removed under reduced pressure and lyophilized to yield 27.26 g PE, 15.48 g CF, 49.49 g EtOAc, and 50.11 g n-BuOH fractions, and 52.13 g aqueous phase residue (AQ). All the samples were kept at −20 °C for further analysis.

### 2.3. Determination of Bioactive Compounds

#### 2.3.1. Total Phenolic Content

The total phenolic content (TPC) was assessed using the Folin-Ciocalteu colorimetric assay [19]. A 40 μL of the properly diluted sample was added to a 96-well microplate and mixed with 20 μL of 0.5 M Folin-Ciocalteu reagents, kept for 5 min at 25 °C. A 160 μL of Na_2_CO_3_ (7.5%, *w*/*v*) was then added and incubated, protected from light, for 30 min at 25 °C. Finally, the absorbance value was measured at 765 nm by a microplate reader (Biotek, VT, USA). Gallic acid (10–100 μg/mL) was used as the standard and results were expressed as μg of gallic acid equivalents (GAE)/mg dry extract (DE).

#### 2.3.2. Total Flavonoid Content

The total flavonoid content (TFC) was measured using a previously reported method [20]. An exact volume of 40 μL of appropriately diluted sample was mixed with 3% NaNO_2_ (20 μL) and kept for 6 min, 20 μL of a 6% Al(NO_3_)_3_ was then added and allowed to react for 6 min, followed by addition of 140 μL of 4% NaOH, and also 60 μL of 70% methanol. The mixture was incubated at room temperature for 15 min and absorbance was determined at a wavelength of 510 nm. Rutin (10–100 μg/mL) was used as the standard reference, and the final result was expressed as μg of rutin equivalents (RE)/mg dry extract (DE).

### 2.4. Determination of Antioxidant Activity

#### 2.4.1. DPPH Radical Scavenging Activity

The DPPH· scavenging activity was quantified by the method reported by Chan et al. [21], with Trolox as the standard. The suitable concentration of sample (100 μL) was mixed with 100 μL 0.15 mM DPPH solution in a 96-well microplate, then the mixtures were kept in darkness for 30 min at room temperature. Subsequently, the absorbance was measured at 517 nm. By plotting the elimination ratio versus the corresponding concentrations of Trolox (20–100 μg/mL), a standard reference was prepared and the DPPH· scavenging activity was calculated. The results were expressed as μg of Trolox equivalents/mg dry extract (DE). Methanol was used as a negative control, whereas ascorbic acid (Vc) and BHT were used as positive controls.

#### 2.4.2. ABTS Radical Scavenging Activity

The ABTS·^+^ scavenging activity was estimated following the reported method [22], with a slight modification. Briefly, ABTS·^+^ solution was prepared by mixing 7 mM ABTS aqueous solution with 2.45 mM potassium persulphate, and incubated in the dark at 25 °C for 12 h to afford an absorbance value of 0.7 ± 0.2 at 734 nm. A 50 μL of the properly diluted sample was added to 200 μL ABTS^+^ solution in a 96-well microplate and incubated at room temperature for 6 min, then the absorbance was measured at 734 nm. Trolox was used as a standard, methanol was used as a negative control, whereas Vc and BHT were used as positive controls. The ABTS·^+^ scavenging activity was expressed as μg of Trolox equivalents/mg dry extract (DE).

#### 2.4.3. Ferric Reducing Antioxidant Power

The ferric reducing antioxidant power (FRAP) was determined according to our previously reported method [23]. FRAP reagent consisted of 7 mM TPTZ, 20 mM FeCl_3_ in acetate buffer (pH 3.6). A 50 μL of the suitable concentration of sample was mixed with 200 μL of FRAP reagent in a 96-well microplate and protected from light at 37 °C. After incubation for 10 min, the absorbance at 593 nm was measured. FeSO_4_ (20–100 μg/mL) was used as a standard, methanol was used as a negative control, whereas Vc and BHT were used as positive controls. The FRAP value was expressed as μg of FeSO_4_ equivalents/mg dry extract (DE).

#### 2.4.4. Superoxide Radical Scavenging Activity

The superoxide anion (O_2_^−^) scavenging activity was examined using a pyrogallol autoxidation method [24]. Briefly, a 20 μL of appropriately diluted sample solution was added to 240 μL Tris-HCl buffer (50 mM, pH 8.2, containing 1 mM Na_2_EDTA) in a 96-well microplate. After incubation in darkness at room temperature for 10 min, 20 μL of pyrogallol (60 mM, prepared in 1 mM HCl) was added. Followed by oscillation for 10 s, the absorbance at 325 nm was measured immediately every minute for 5 min, and the slope of the absorbance vs. time plot was calculated. The superoxide radical scavenging activity of samples were defined as the degree of autoxidation rate reduction. Trolox was used to generate a standard reference curve and results were expressed as mg Trolox equivalents/g dry extract (DE). Methanol was used as the negative control, whereas Vc and BHT were used as the positive controls.

### 2.5. Determination of Enzymes Inhibitory Activity

#### 2.5.1. α-Glucosidase Inhibitory Ability

The α-glucosidase inhibitory ability was assessed as our previous study [15]. A 50 μL of the suitable concentration of sample was mixed with 20 μL of 0.1 U/mL α-glucosidase in a black 96-well microplate, then 50 μL of 84 μM freshly prepared 4-MUG solution was added and incubated in darkness for 20 min at 37 °C. Finally, the reaction was stopped by the addition of 100 μL of 100 mM glycine-NaOH buffer (pH 10.6). Followed by shaking on an orbital shaker for 30 s, the fluorescence value at λ_ex_ 355 nm and λ_em_ 460 nm was measured. Methanol and acarbose were negative and positive controls, respectively. Results were expressed as IC_50_ value (μg/mL), which was calculated by non-linear regression analysis between the percentage of inhibition and sample concentration.

#### 2.5.2. Acetylcholinesterase Inhibitory Ability

The acetylcholinesterase (AChE) inhibitory properties of the extracts were tested using a 96-well microplate colorimetric method [25]. A 50 μL of the suitable concentration of sample was mixed with 90 μL of Ellman’s solution (containing 15 μL of 15 mM ATCI and 75 μL of 3 mM DTNB) in a 96-well microplate and incubated in darkness for 10 min at 30 °C. Then 20 μL of 0.2 U/mL AChE was added and kept in darkness for 5 min. Finally, the absorbance value at 405 nm was measured. Methanol and galanthamine were negative and positive controls, respectively. The AChE inhibitory ability was expressed as IC_50_ value (mg/mL).

#### 2.5.3. Tyrosinase Inhibitory Ability

The tyrosinase inhibitory ability was determined according to the reported method [26], with little modification. First, 50 μL of the suitable concentration of the sample was added to 150 μL of mushroom tyrosinase solution (100 U/mL) and incubated for 15 min at 37 °C, followed by 50 μL of 2.5 mM L-dopa was added and reacted at 37 °C for 10 min, the absorbance value was measured at 475 nm. Methanol was used as the solvent control, while kojic acid was used as the positive control, and results were expressed as IC_50_ value (μg/mL).

### 2.6. Determination of Anticancer Activity

The inhibition on HepG2 and MCF-7 cell proliferation was measured by using the CCK-8 assay according to our previous report [15]. Cells were seeded in 96-well plates (5 × 10^3^ cells per well) and placed in a cell culture incubator at 37 °C. After 24 h of incubation, a 10 μL of the suitable concentration of sample was added and kept for 24 h, followed by the addition of CCK-8 solution (10 μL), cells were incubated for a further 4 h, the absorbance at 450 nm was measured. Cisplatin was used as a positive control. The cell proliferation inhibitory ability was expressed as the IC_50_ value (μg/mL). In addition, the cytotoxic effect was also evaluated by measuring the viability of cells after treatment with the sample for 48 h.

### 2.7. HPLC-DAD Analyses

HPLC-DAD analysis was performed by an HPLC 1260 (Agilent Technologies, CA, USA) equipped with a degasser, quaternary pump solvent delivery, thermo-stated column compartment, and a diode array detector (DAD). Chromatographic separations were performed on the C18 reversed-phase analytical column (250 mm × 4.6 mm, 5 μm, Greenherbs Science and Technology) at 25 °C, while 0.1% formic acid (A) and acetonitrile (B) were used as the mobile phases with a flow rate of 0.8 mL/min. The gradient elution conditions of the mobile phase B were: 0–12 min, 2–8%; 12–15 min, 8–13%; 15–30 min, 13–18%; 30–50 min, 18–30%; 50–60 min, 30–50%; 60–70 min, 50–70%; 70–80 min, 70–90%; 80–85 min, 90–100%; 85–90 min, 100–2%. Samples were filtered through nylon syringe filters (0.22 μm) and injected in triplicate with an injection volume of 20 μL. The chromatogram was monitored from 200 to 400 nm and the peak areas were calculated. Finally, compounds were identified and quantified by comparison with the retention times and peak areas from standards. In this study, five-point calibration curves were prepared using gallic acid (y = 662.72x + 73.856, *R*^2^ = 0.9995) at 280 nm, catechin (y = 185.86x − 26.949, *R*^2^ = 0.9991) at 280 nm, chlorogenic acid (y = 336.05x − 1.1845, *R*^2^ = 0.9998) at 310 nm, L-epicatechin (y = 213.05x + 16.625, *R*^2^ = 0.9983) at 280 nm, dihydromyricetin (y = 446.13x + 23.478, *R*^2^ = 0.9994) at 280 nm, rutin (y = 128.39x + 0.3309, *R*^2^ = 0.9972) at 360 nm, ferulic acid (y = 484.56x + 1.9186, *R*^2^ = 0.9997) at 340 nm, (-)-epicatechin gallate (y = 413.08x + 89.615, *R*^2^ = 0.9954) at 280 nm, rosmarinic acid (y = 274.64x − 27.312, *R*^2^ = 0.9993) at 310 nm, baicalin (y = 324.12x − 1.6326, *R*^2^ = 0.9974) at 310 nm, luteolin (y = 20.373x − 1.2548, *R*^2^ = 0.9963) at 360 nm, apigenin (y = 472.77x − 0.899, *R*^2^ = 0.9993) at 340 nm, hesperetin (y = 247.1x + 15.53, *R*^2^ = 0.9992) at 310 nm, baicalein (y = 239.15x − 31.549, *R*^2^ = 0.9985) at 340 nm, myricetin (y = 226.05x − 7.4156, *R*^2^ = 0.9965) at 360 nm, quercetin (y = 264.13x + 8.0285, *R*^2^ = 0.9995) at 360 nm, and resveratrol (y = 289.2x − 5.31, *R*^2^ = 0.9998) at 340 nm, where x was concentration, and y was area peak, and the concentration of all samples ranged varied from 20 to 100 μg/mL. The results were expressed as μg equivalents of the corresponding standard per mg of extract.

### 2.8. Determination of Antidiabetic Activity

#### 2.8.1. Animals and Treatments

The animal rats were purchased from Hunan SJA Laboratory Animal Co., Ltd. (Changsha, Hunan) and the production certificate number and use license number are SCXK (Hunan) 2016-0002 and SYXK (Hunan) 2016-0002, respectively. All animal experiments were done in the animal laboratories of Southwest Forestry University (Kunming, Yunnan), and adhered to the guidelines for care and use of laboratory animals, and approved by the Academic Committee of Southwest Forestry University (ethical approval number SWFU-2018003). In this study, the T2DM rat model was induced by the method described previously [27] with some modifications. Briefly, thirty specific-pathogen-free Sprague–Dawley (SD) male rats (8-week-old, 200 ± 20 g) were housed in a room with a constant temperature (23 ± 2 °C) and humidity (55 ± 5%), one per cage, under a 12 h light/dark cycle with free access to food and water. After a week of adaptation, the model groups were fed a high-fat diet (TP23400, 60% fat, 14.1% protein, and 25.9% carbohydrate, TROPHIC Animal Feed High-Tech Co. LTD, China) for 4 weeks, while the normal group was fed a normal diet (TP23402, 10% fat, 14.1% protein, and 75.9% carbohydrate). Thereafter, the model groups were given an intraperitoneal injection of a low single dose of a freshly prepared STZ solution (45 mg/kg in 0.1 M citrate buffer; pH 4.0) after fasting for 12 h, the normal group received an equal volume of citrate buffer. Three days after injection, the level of blood glucose was measured via the tail vein with a blood glucose meter (OneTouch Ultra, Johnson & Johnson, USA) and rats with glucose concentration >16.7 mmol/L were considered as type *2* diabetic rats. After successful modeling, the rats were randomly divided into five groups (6 rats per group): normal diet (ND), high-fat diet (HFD), high-fat diet with medium dose extracts (HFD-ME), high-fat diet with high dose extracts (HFD-HE), and high-fat diet with acarbose (HFD-A). The *P. frutescens* leaf extract fraction with the strongest α-glucosidase inhibitory activity and acarbose were added to feed and mixed thoroughly, and used as the treatment and reference drug respectively. The HFD-ME and HFD-HE groups were fed with the mixed diet with the extract fractions dose of 100 and 250 mg/kg body weight (BW) per day respectively, whereas the HFD-A group were received acarbose (100 mg/kg BW per day). The HFD and ND group were continued to feed with a high-fat diet and a normal diet, respectively. The administration period was 4 weeks, and BW, food and water intake, and postprandial blood glucose were recorded weekly.

#### 2.8.2. Blood and Tissue Sample Collection

At the end of the experiment, after 12 h of fasting, the rats were sacrificed after being sedated with anesthesia, blood was collected from the heart and centrifuged at 4000× *g* for 10 min at 4 °C, and the supernatant was collected and stored at −80 °C for subsequent analyses. The total cholesterol (TC), triglyceride (TG), low-density lipoprotein cholesterol (LDL-C), and high-density lipoprotein cholesterol (HDL-C) levels were determined using an automatic biochemical analyzer (Abbott Laboratories, Abbott Park, Green Oaks, IL, USA). The liver and intestinal tissue were fixed in 4% paraformaldehyde solution, and embedded in paraffin and cut into 5-μm sections for hematoxylin and eosin (H&E) staining and periodic acid-Schiff (PAS) staining, and assessed by light microscopy.

### 2.9. Statistical Analysis

All experiments were repeated three times to obtain the mean values (mean ± standard deviation). Statistical analyses were carried out using SPSS 20.0 software (IBM, Armonk, NY, USA). Tukey’s test and one-way analysis of variance were used to find significant differences, and a two-tailed Pearson test was used to analyze the correlation between different factors. *p* < 0.05 was considered as significant (*), and <0.01 as highly significant (**). Principal component analysis (PCA) was performed using R (ver 3.6.3).

## 3. Results

### 3.1. Total Phenolic and Flavonoid Content

According to the previous report, *P. frutescens* leaves were found to be rich in phenolic and flavonoid components [28], which were the important secondary metabolites of many plants and exhibited a variety of biochemical and biological activities. The TPC and TFC of *P. frutescens* leave crude extract and the different fractions used in this study are displayed in Table 1. The results showed that TPC was highest in the crude extract (CE), followed by the ethyl acetate fraction (EtOAc), n-butanol fraction (n-BuOH), chloroform fraction (CF), petroleum ether fraction (PE), and finally, the aqueous phase residue (AQ). The highest TFC was detected in the EtOAc, followed by the CE, CF, n-BuOH, AQ, and PE. The phenolic and flavonoid concentrations differed greatly in various fractions (from 86.45 ± 18.82 to 440.48 ± 13.08 μg of GAE/mg DE, and from 85.68 ± 18.82 to 455.22 ± 61.03 μg of RE/mg DE, respectively), which depended on the solvent polarity.

### 3.2. Antioxidant Properties

In vitro antioxidant effect of *P. frutescens* leaf extract and its fractions were examined using four different assays (DPPH, ABTS^+^, FRAP, and superoxide anion) and the results are depicted in Table 2. Among all the four antioxidant assays, EtOAc fraction displayed the best antioxidant activity and was significantly greater than the crude extract and other fractions (*p* < 0.05), with the values of 1006.33 ± 15.80, 1682.80 ± 38.49, and 1957.73 ± 101.86 μg of Trolox/mg DE for DPPH, ABTS^+^, and superoxide anion scavenging activity, respectively. For FRAP, it was 4181.13 ± 324.28 μg of FeSO_4_/mg DE. This is consistent with the results of previous study [29]. Surprisingly, EtOAc fraction even exhibited stronger antioxidant activity than Vc and BHT, the two most commonly used synthetic antioxidants. In addition, CE, CF, and n-BuOH fractions also possessed better superior antioxidant activities than other fractions and BHT.

**Figure 1:**
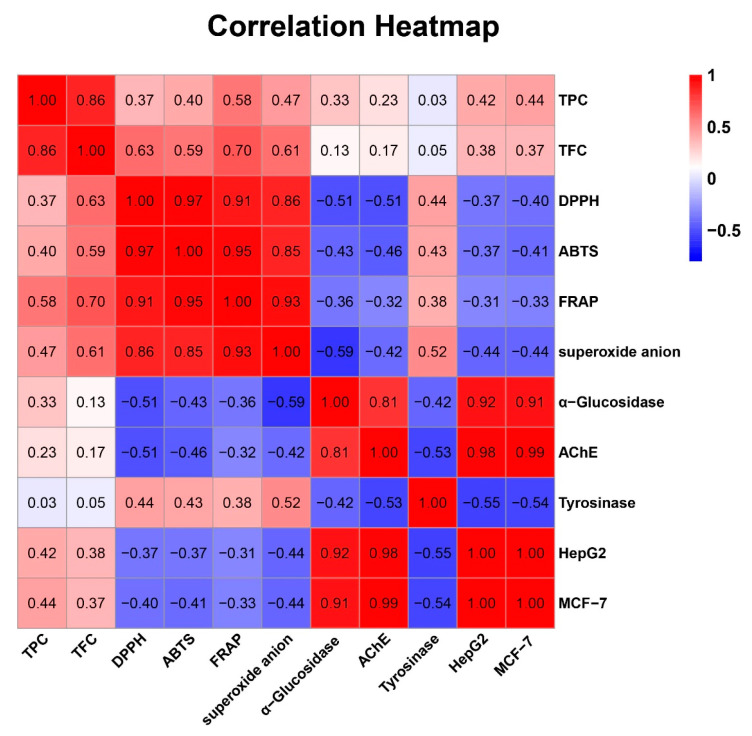
Results of correlational analyses from experiment. The Pearson coefficient (*r*) is given for each correlation in the corresponding color, and the *p* value represented as a heatmap ranging from −1 to 1 (red to blue). TPC = total phenolic content; TFC = total flavonoid content; DPPH = DPPH·scavenging activity; ABTS = ABTS·^+^ scavenging ability; FRAP = ferric reducing antioxidant power; superoxide anion = superoxide anion scavenging activity; α-Glucosidase = the IC_50_ value for α-glucosidase inhibitory; AChE = the IC_50_ value for acetylcholinesterase inhibitory activity; Tyrosinase = the IC_50_ value for tyrosinase inhibitory; HepG2 = the IC_50_ value for inhibition on HepG2 cell proliferation; MCF-7 = the IC_50_ value for inhibition on MCF-7 cell proliferation.

**Figure 2:**
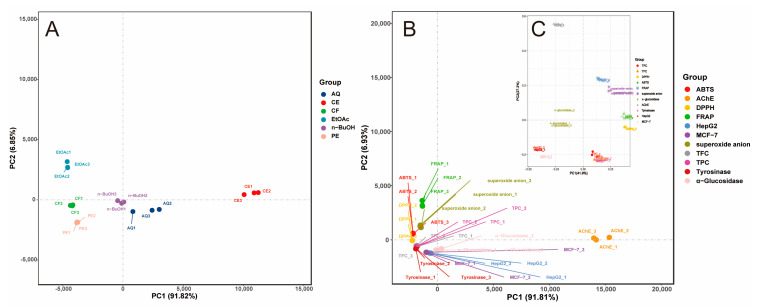
Principal component analysis (PCA) of different samples and indicators. (**A**) PCA of six *P. frutescens* leaf extract and fractions; (**B**) PCA of 11 indicators of active ingredient content and biological activity; (**C**) principal co-ordinates analysis (PCoA) of 11 indicators of active ingredient content and biological activity. CE = crude extract; PE = petroleum ether fraction; CF = chloroform fraction; EtOAc = ethyl acetate fraction; n-BuOH = n-butanol fraction; AQ = aqueous phase residue; TPC = total phenolic content; TFC = total flavonoid content; DPPH = DPPH· scavenging activity; ABTS = ABTS^+^ scavenging ability; FRAP = ferric reducing antioxidant power; superoxide anion = superoxide anion scavenging activity; α-Glucosidase = the IC_50_ value for α-glucosidase inhibitory; AChE = the IC_50_ value for acetylcholinesterase inhibitory activity; Tyrosinase = the IC_50_ value for tyrosinase inhibitory; HepG2 = the IC_50_ value for inhibition on HepG2 cell proliferation; MCF-7 = the IC_50_ value for inhibition on MCF-7 cell proliferation.

Pearson correlation analysis (Figure 1) revealed that FRAP and superoxide anion scavenging activity had significant correlations with TPC (*r* = 0.582 and 0.469, *p* < 0.05), and strong correlations with TFC (*r* = 0.695 and 0.612, *p* < 0.01). DPPH·scavenging and ABTS·^+^ scavenging activity were not significantly associated with TPC, but highly correlated with TFC (*r* = 0.628, *p* < 0.01, and *r* = 0.587, *p* < 0.05, respectively). Results of principal component analysis (PCA) are shown in Figure 2, these four antioxidant activities had almost identical principal component coordinates and relatively close distances to TPC and TFC, and they all could be well differentiated by principal coordinates analysis (PCoA). For different extracts and fractions, the PCA results were also easily distinguishable. Thus, the presence of phenolic and flavonoid compounds are the main contributors to antioxidant activities, and the difference between these correlations might be due to the different mechanisms of the antioxidant actions, such as hydrogen atom, single electron transfer, and metal chelation [15].

### 3.3. Enzymes Inhibitory Activity

It is believed that α-glucosidase, acetylcholinesterase (AChE), and tyrosinase are important metabolic enzymes relating with oxidative stress, and they are shown to be the key targets of T2DM, Alzheimer’s disease (AD), mammalian melanogenesis, and fruit or vegetable enzymatic browning, respectively [30,31,32]. Natural food and medicinal plants are excellent resources of α-glucosidase, AChE, and tyrosinase inhibitors, and have the potential to be novel strategies for the prevention and treatment of these diseases or hazards [32,33,34].

**Figure 3:**
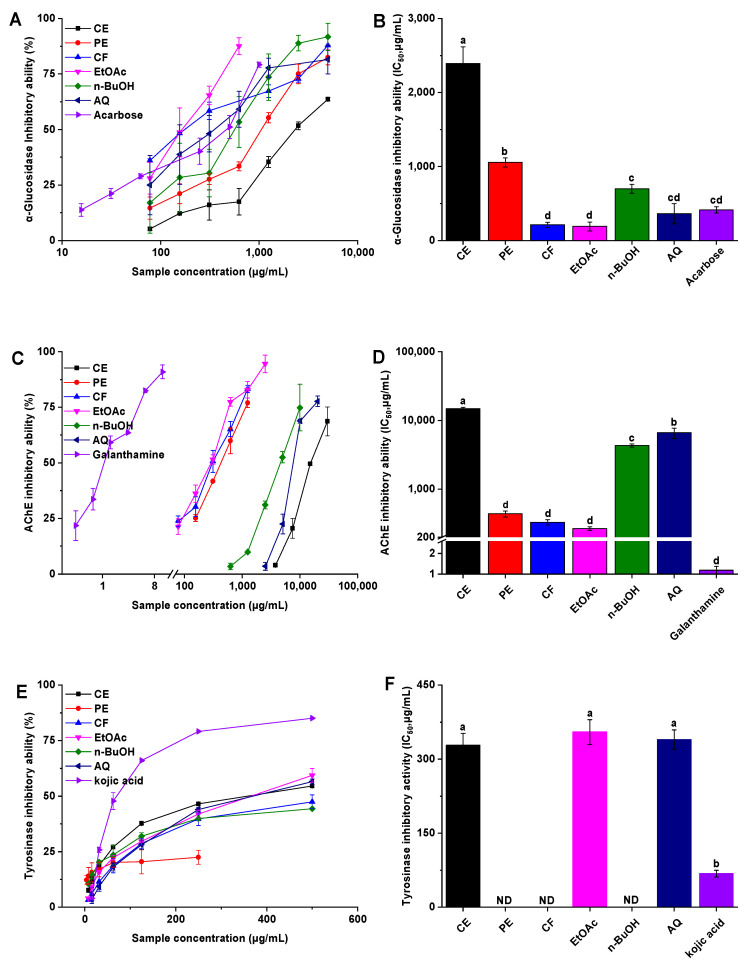
The enzymes inhibitory activities of the *P. frutescens* leaf extract and its fractions. (**A**) The inhibition ratio of α-glucosidase incubated with different concentrations of samples; (**B**) the IC_50_ values of different samples for α-glucosidase inhibition; (**C**) the inhibition ratio of acetylcholinesterase incubated with different concentrations of samples; (**D**) the IC_50_ values of different samples for acetylcholinesterase inhibition; (**E**) the inhibition ratio of tyrosinase incubated with different concentrations of samples; (**F**) the IC_50_ values of different samples for tyrosinase inhibition. ND = not detected; CE = crude extract; PE = petroleum ether fraction; CF = chloroform fraction; EtOAc = ethyl acetate fraction; n-BuOH = n-butanol fraction; AQ = aqueous phase residue. Bars with different letters indicate a significant difference (*p* < 0.05).

The inhibition rates of *P. frutescens* leaf extract and its fractions against α-glucosidase are shown in Figure 3A, and the IC_50_ values are presented in Figure 3B. All samples demonstrated certain α-glucosidase inhibitory activity in a concentration-dependent manner. Among them, the EtOAc and CF fractions showed the strongest effect on inhibiting α-glucosidase, followed by AQ, n-BuOH, PE, and CE. Delightedly, and the IC_50_ values of the EtOAc, CF, and AQ were 190.03 ± 61.29, 212.86 ± 33.98, and 362.57 ± 136.99 μg/mL, which were superior to control acarbose (414.46 ± 42.22 μg/mL), respectively. Correlation analyses revealed that α-glucosidase inhibitory activity was significantly negatively associated with DPPH· scavenging activity and superoxide anion scavenging activity (Figure 1). Considering the postprandial blood glucose levels could be reduced by inhibiting the activity of α-glucosidase not only in vitro but also in vivo, the EtOAc fraction was selected for the subsequent animal experiments to evaluate the hypoglycemic effect [35].

In Figure 3C,D, similar to the results of the inhibition of α-glucosidase activity, *P. frutescens* leaf extract and different fractions showed AChE inhibitory activity in a dose-dependent manner. Overall, the EtOAc (IC_50_ value was 267.67 ± 17.15 μg/mL), CF (IC_50_ value was 329.77 ± 29.62 μg/mL), and PE (IC_50_ value was 438.68 ± 44.11 μg/mL) fractions performed significantly better inhibition activity than the other fractions (IC_50_ value ranging from 4332.67 ± 233.81 to 14924.08 ± 605.75 μg/mL). According to the results of correlation analysis, AChE inhibitory activity was significantly negatively associated with DPPH· scavenging activity (*r* = −0.51, *p* < 0.05), and strong correlation with α-glucosidase inhibitory activity (*r* = 0.81, *p* < 0.01), while correlations with TPC, TFC, FRAP, and superoxide anion scavenging activity were not significant.

The results shown in Figure 3E,F indicated that CE, AQ, and EtOAc fractions had activity against tyrosinase with the IC_50_ value of 328.04 ± 24.16, 339.61 ± 19.74, and 354.84 ± 25.30 μg/mL, whereas no inhibitory activity was detected for other fractions.

According to Figure 2B,C, although α-glucosidase, AChE, and tyrosinase inhibitory activities did not yield a complete group separation on the PCA2 axes, they clustered far from each other along the PCA1 axis. Of which, α-glucosidase and AChE inhibitory activities were separated from TPC, TFC, and showed a substantial distance with four antioxidant activities. These results suggested that the bioactivities might be interrelated but not the main influence factors for each other, which could be due to the multicomponent and multitarget characteristics of *P. frutescens* leaf extract.

### 3.4. Anticancer Activity

Recent studies reported that diabetes could contribute to the development of cancer via inflammation, and thus has been identified as a risk factor for cancer and cancer-related mortality [36,37]. Malignant tumors such as hepatocellular carcinoma and breast cancer are important causes of human death, and a significant link between them and diabetes has been evidenced [38,39,40,41].

**Figure 4:**
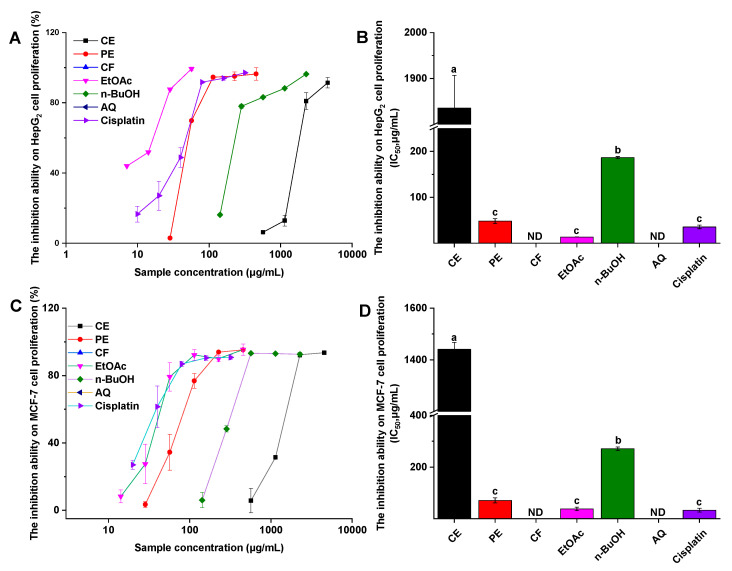
The cell proliferation inhibitory activity of the *P. frutescens* leave extract and its fractions. (**A**) The inhibition ratio of HepG2 cell proliferation incubated with different concentrations of samples; (**B**) the IC_50_ values of different samples for HepG2 cell proliferation inhibition; (**C**) the inhibition ratio of MCF-7 cell proliferation incubated with different concentrations of samples; (**D**) the IC_50_ values of different samples for MCF-7 cell proliferation inhibition. ND = not detected; CE = crude extract; PE = petroleum ether fraction; CF = chloroform fraction; EtOAc = ethyl acetate fraction; n-BuOH = n-butanol fraction; AQ = aqueous phase residue. Bars with different letters indicate a significant difference (*p* < 0.05).

In this study, the inhibition of the *P. frutescens* leaf extract and its fractions on HepG2 and MCF-7 cell growth were performed to evaluate its anticancer activities. From Figure 4, only EtOAc, PE, n-BuOH, and CE fractions showed inhibitory effects on HepG2 and MCF-7 cell proliferation in a dose-dependent manner. The inhibition activity of these fractions on HepG2 cell are shown in Figure 4B, and the IC_50_ values of EtOAc, PE, n-BuOH, and CE were 13.43 ± 0.10, 48.07 ± 5.27, 186.43 ± 2.39, and 1835.73 ± 71.05 μg/mL, respectively, while it was 35.53 ± 3.89 μg/mL for the control cisplatin. No significant difference was found between EtOAc and PE (*p* > 0.05). According to the similar results presented in Figure 4D, EtOAc, PE, n-BuOH, CE, and cisplatin inhibited MCF-7 cell with IC_50_ values of 37.92 ± 6.82, 70 ± 19.74, 271.26 ± 6.80, 1439.89 ± 27.70, and 33.03 ± 7.49 μg/mL, respectively. Similarly, there was no significant difference between EtOAc, PE, and cisplatin.

From Figure 1, for the *P. frutescens* leaf extract and its fractions, their capacities to inhibit HepG2 cell proliferation were significantly correlated with their abilities to inhibit MCF-7 cell (*r* = 1.00, *p* < 0.01), α-glucosidase (*r* = 0.92, *p* < 0.01), and AChE (*r* = 0.98, *p* < 0.01). Likewise, the MCF-7 cell proliferation inhibitory ability was significantly associated with α-glucosidase inhibitory activity (*r* = 0.91, *p* < 0.01) and AChE inhibitory activity (*r* = 0.99, *p* < 0.01). In light of the findings from PCA results (Figure 2), TPC, TFC, α-glucosidase and AChE inhibitory activity, and anticancer activity demonstrated closer relationship between each other along the PC2 axis. These combined findings suggested that α-glucosidase and AChE inhibitory activity, and anticancer activity, may be due to the combined effects of the complex ingredients in *P. frutescens* leaves, rather than a single component.

### 3.5. HPLC-DAD Analyses

As in the preceding sections, the EtOAc fraction presented high active ingredient content and the strongest biological activities in all fractions, thus it was selected for further analyses. In this study, the components of the EtOAc fraction were analyzed by HPLC-DAD. Five phenolic acids and nine flavonoids were identified and quantified, and detailed results are displayed in Table 3. Of these, the content of luteolin, rosmarinic acid, rutin, and catechin were 254.56 ± 3.74, 163.88 ± 2.53, 97.56 ± 11.09, and 94.70 ± 1.35 μg/mg, which were the dominant components, respectively. Additionally, gallic acid, chlorogenic acid, L-epicatechin, dihydromyricetin, ferulic acid, (-)-epicatechin gallate, baicalin, apigenin, hesperetin, and baicalein were also detected. Many previous studies described the presence of these compounds in *P. frutescens* leaves crude extract and their biological activities, such as antioxidant, α-glucosidase inhibition, anticancer, and hypoglycemic activity [28,29,42,43].

### 3.6. Antidiabetic Activity

#### 3.6.1. Physical Appearance and Biochemical Parameters

**Figure 5:**
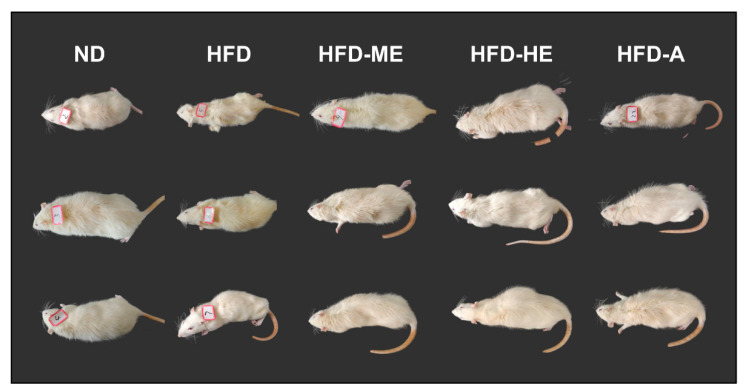
Effect of the ethyl acetate fraction of *P. frutescens* leaves on the appearance of STZ-induced diabetic rats. ND = normal diet group; HFD = high-fat diet group; HFD-ME = high-fat diet with medium dose extracts group; HFD-HE = high-fat diet with high dose extracts group; HFD-A = high-fat diet with acarbose group.

**Figure 6:**
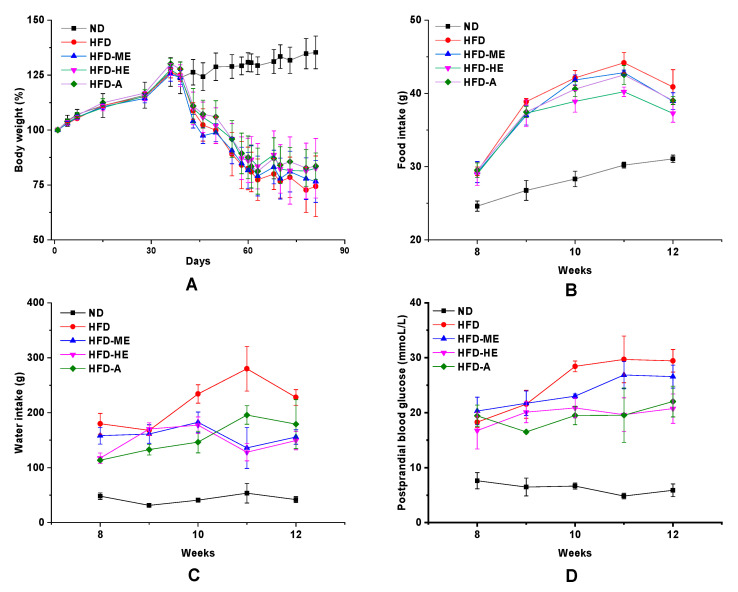
Effect of the ethyl acetate fraction of *P. frutescens* leaves on the biochemical parameters of STZ-induced diabetic rats. (**A**) Body weight; (**B**) food intake; (**C**) water intake; (**D**) postprandial blood glucose. ND = normal diet group; HFD = high-fat diet group; HFD-ME = high-fat diet with medium dose extracts group; HFD-HE = high-fat diet with high dose extracts group; HFD-A = high-fat diet with acarbose group.

As shown in Figure 5, STZ-induced diabetic rats showed symptoms of mental depression, lusterless body complexion, and slow response. In addition, diabetic groups exhibited increasing blood glucose levels, food intake, water intake, and decreased bodyweight, which were the important hallmarks of T2DM (Figure 6) [44]. After 4 weeks of administration with the EtOAc fraction of *P. frutescens* leaves, these appearances were significantly improved. Treated rats had soft and shiny hair, and were shown to be significantly more sensitive to the reaction (Figure 5).

There were no statistically significant differences in the body weight between HFD, HFD-ME, HFD-HE, and HFD-A groups, but showed a significant reduction when compared with the ND group at *p* < 0.05 (Figure 6A). From Figure 6, the consumption of EtOAc fraction and acarbose reduced food intake, water intake, and postprandial glucose concentrations. Surprisingly, the ameliorative potential of high-dose EtOAc fraction was equivalent to or better than that of acarbose, a well-known commercial drug for diabetes.

Dyslipidemia commonly occurs in metabolic syndrome disease patients such as diabetics, therefore, the beneficial effects on glucose metabolism were also associated with an improvement in lipid profile [45,46]. To determine the effect of EtOAc fraction on dyslipidemia, the lipid levels in the rat serum were measured, and the results are shown in Table 4. The HFD group showed a significant increase in TC, TG, and LDL-C, while a significant decrease in HDL-C compared to the NC group was seen. Feeding of EtOAc fraction significantly reduced the TC, TG, and LDL-C levels, and increased the HDL-C levels of HFD-fed rats (*p* < 0.05); here, the high-dose treatment group was significantly superior to the positive control acarbose group (*p* < 0.05).

#### 3.6.2. Histopathology Changes of the Liver and Intestinal

**Figure 7:**
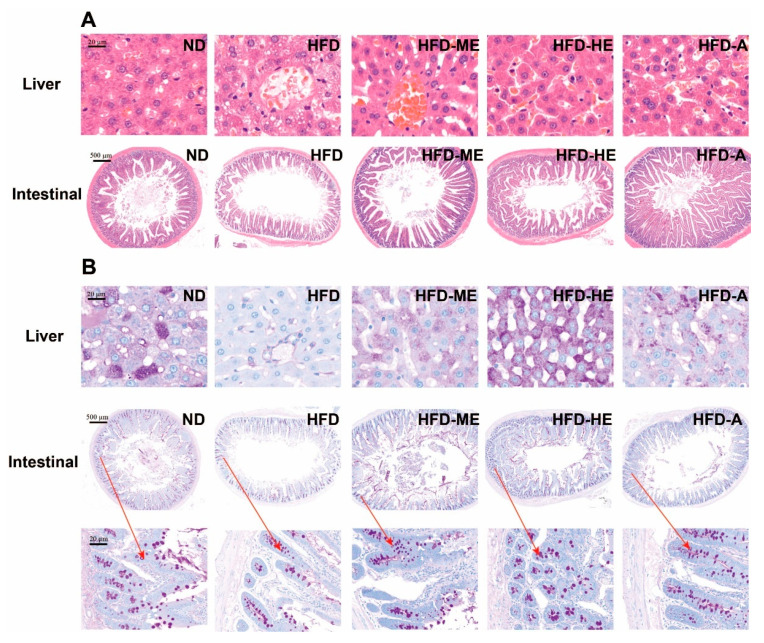
Effect of the ethyl acetate fraction of *P. frutescens* leaves on the histopathology of STZ-induced diabetic rats. (**A**) H&E staining; (**B**) PAS staining. ND = normal diet group; HFD = high-fat diet group; HFD-ME = high-fat diet with medium dose extracts group; HFD-HE = high-fat diet with high dose extracts group; HFD-A = high-fat diet with acarbose group.

The liver and intestinal were the main tissue involved in energy and glucose metabolism [47,48]. Histological analysis of the liver and intestinal tissues by H&E staining are shown in Figure 7A, and the microscope pictures show the transverse sections of tissues. The hepatocytes of the ND group had a clear morphology, round or oval cell clusters, centrally located nuclei, and clear boundaries, while that of diabetic rats showed marked vacuolization, reducing nuclei size, and disordered liver structure. The rats treated with EtOAc fraction and acarbose showed improvement in the histological abnormalities with an obvious increase in the number of hepatocytes and improved shape. The organization of the intestinal tissue of rats in the ND group was normal, and the mucosal villous structure was neatly arranged with a clear structure, while the intestinal lesions of diabetic rats were obvious, and some intestinal villi were necrotic and shed or even disappeared. Average villus length was increased at varying degrees throughout the intestine in EtOAc fraction and acarbose-treated groups compared with the ND group.

Glycogen is the polyglucosan storage and PAS staining is used as a qualitative indicator of glycogen accumulation and cell damage [49]. Figure 7 indicated that the ND group showed rich glycogen storage in the liver and intestinal tissues, while the ND group showed a decrease in the accumulation of glycogen, which might account for the diabetes-induced reductions in the capability of synthesizing and accumulating glycogen [50]. After 4 weeks of treatments with the EtOAc fraction, the glycogen accumulation in the liver and intestinal was effectively increased. Compared to acarbose, the high dose of the EtOAc fraction showed a stronger ability to promote glycogen synthesis, and then controlled hyperglycemia.

## 4. Discussion

It is known that there are close relationships between oxidative stress and the development of various diseases such as diabetes mellitus, cancer, neurodegenerative disease, and metabolic syndrome [51,52]. Many active ingredients in traditional food and medicinal plant have shown excellent effects in the prevention of oxidative stress-induced health risks, and this phenomenon has become a key focus of the current study [53]. Because of the complexity of these illnesses, and the multi-component system in food/plants, most researches are limited to the impacts of the entire extract on one/few indexes of oxidative stress and its corresponding metabolic diseases, there are relatively fewer studies on the effects of the subdivided components of these food/plants on multiple oxidative stress-related diseases.

As an important medicine food plant in South East Asia, *P. frutescens* has been shown to have rich biochemical compounds and many biological activities including the antidiabetic activity in vitro, but few studies have been conducted on its hypoglycemic potential in vivo. In this study, *P. frutescens* leaves were extracted using methanol solution and isolated by liquid–liquid partitioning, and the functional activities and chemical compositions of different polar fractions were investigated.

Results showed that the EtOAc fraction possessed higher total phenolic content and the highest total flavonoid content. It also showed the strongest DPPH radical, ABTS radical, superoxide anion scavenging activity, and ferric reducing antioxidant power, even stronger than Vc and BHT. The α-glucosidase inhibition potency of each fraction was evaluated, and the EtOAc fraction displayed the most potent α-glucosidase inhibitory activity twice higher than acarbose, and the same trend was reflected in the AChE inhibition experiment. Additionally, the EtOAc fraction has the highest inhibitory effect on MCF-7 and HepG2 cell proliferation. Considering that there was a strong correlation with each other between these in vitro activities and oxidative stress-related diseases, the EtOAc fraction was used for HPLC analysis and the subsequent animal experiment.

The HPLC-DAD analysis of the EtOAc fraction detected five phenolic acids and nine flavonoids, and luteolin, rosmarinic acid, rutin, and catechin were the dominant components of the fraction. To assess the hypoglycemic effect of the EtOAc fraction, the T2DM SD rat model induced by high-fat diet/low-dose STZ was established, and the physical appearance, biochemical parameters, and histopathology changes were evaluated. Results showed that the EtOAc fraction could significantly decrease the serum glucose level, food and water intake, and increased the body weight of diabetic rats, and improved their serum levels of TC, TG, HDL-C, and LDL-C.

Histopathological analysis of the livers and intestinal tissue revealed EtOAc fraction treatments could effectively ameliorate diabetes-related pathophysiology conditions in livers and intestinal tissue, such as tissue injury and villus rupture. PAS staining analysis showed that the intake of EtOAc fraction could increase glycogen accumulation in the livers and intestine, and thereby avoid the entrance of glucose into the bloodstream, which in turn caused hyperglycemia and diabetes.

Taken together, the EtOAc fraction of *P. frutescens* leaves exhibited excellent antioxidant activity, α-glucosidase, acetylcholinesterase, tyrosinase inhibition activity, anticancer activity, and in vivo hypoglycemic activity.

## 5. Conclusions

In this study, *P. frutescens* leaves showed promising hypoglycemic activity in vitro and in vivo, which could be exploited as a source of natural antidiabetic. To the best of our knowledge, this is the first comparative study of the biological activities of different solvent extracts of *P. frutescens* leaves, and demonstrate the beneficial effect of its EtOAc fraction in hypoglycemic actions. Further research is needed to investigate the underlying mechanisms of the hypoglycemic effects by researching the transcriptomics, metabolomics, and intestinal microbiota, etc.

## Figures and Tables

**Table 1:** Total phenolic and flavonoid content in *P. frutescens* leaf extract and its fractions.

Samples	TPC (μg of GAE/mg DE)	TFC (μg of RE/mg DE)
CE	450.83 ± 11.74 ^a^	411.69 ± 43.50 ^a^
PE	200.34 ± 6.51 ^c^	85.68 ± 18.82 ^c^
CF	281.31 ± 8.57 ^b^	384.24 ± 3.53 ^a,b^
EtOAc	440.48 ± 13.08 ^a^	455.22 ± 61.03 ^a^
n-BuOH	419.78 ± 48.88 ^a^	307.86 ± 12.10 ^b^
AQ	86.45 ± 18.82 ^d^	104.00 ± 31.69 ^c^

TPC = total phenolic content; TFC = total flavonoid content; CE = crude extract; PE = petroleum ether fraction; CF = chloroform fraction; EtOAc = ethyl acetate fraction; n-BuOH = n-butanol fraction; AQ = aqueous phase residue. The different letters represent a significant difference, *p* < 0.05.

**Table 2:** The antioxidant activities of the *P. frutescens* leaf extract and its fractions.

Samples	DPPH Scavenging Activity (μg of Trolox/mg DE)	ABTS^+^ Scavenging Activity (μg of Trolox/mg DE)	FRAP (μg of FeSO_4_/mg DE)	Superoxide Anion Scavenging Activity (μg of Trolox/mg DE)
CE	93.82 ± 1.92 ^c^	184.77 ± 4.71 ^e^	873.79 ± 30.47 ^d^	440.92 ± 33.51 ^e^
PE	57.32 ± 5.59 ^d^	194.92 ± 1.37 ^e^	150.24 ± 14.59 ^d^	235.14 ± 72.32 ^f^
CF	562.16 ± 23.54 ^b^	581.70 ± 10.93 ^d^	1216.90 ± 71.32 ^c^	977.74 ± 66.28 ^c^
EtOAc	1006.33 ± 15.80 ^a^	1682.80 ± 38.49 ^a^	4181.13 ± 324.28 ^a^	1957.73 ± 101.86 ^a^
n-BuOH	94.82 ± 15.80 ^c^	134.46 ± 27.72 ^e^	1256.56 ± 122.21 ^c^	1114.23 ± 28.19 ^c^
AQ	84.43 ± 1.12 ^c,d^	118.37 ± 2.64 ^e^	353.59 ± 36.83 ^d^	675.53 ± 48.04 ^d^
Vc	588.68 ± 10.54 ^b^	1479.39 ± 59.27 ^b^	1741.74 ± 23.20 ^b^	1513.92 ± 21.72 ^b^
BHT	87.25 ± 5.84 ^c,d^	922.84 ± 23.12 ^c^	784.15 ± 7.73 ^d^	247.19 ± 10.72 ^f^

CE = crude extract; PE = petroleum ether fraction; CF = chloroform fraction; EtOAc = ethyl acetate fraction; n-BuOH = n-butanol fraction; AQ = aqueous phase residue. The different letters represent a significant difference, *p* < 0.05.

**Table 3:** The HPLC analysis of the ethyl acetate fraction of *P. frutescens* leaves.

Retention Time (min)	Compounds	Types of Compounds	Measuring Wavelength (nm)	Content (μg/mg)
9.99	Gallic acid	Phenolic acids	280	11.13 ± 0.23 ^e,f^
16.78	Catechin	Flavonoids	280	94.70 ± 1.35 ^c^
22.40	Chlorogenic acid	Phenolic acids	310	14.09 ± 2.11 ^e^
23.46	L-Epicatechin	Phenolic acids	280	14.41 ± 0.26 ^e^
27.22	Dihydromyricetin	Flavonoids	280	3.71 ± 0.41 ^f^
30.21	Rutin	Flavonoids	360	97.56 ± 11.09 ^c^
36.71	Ferulic acid	Phenolic acids	340	3.78 ± 0.06 ^f^
39.51	(-)-Epicatechin gallate	Flavonoids	280	2.11 ± 0.69 ^f^
40.25	Rosmarinic acid	Phenolic acids	310	163.88 ± 2.53 ^b^
43.86	Baicalin	Flavonoids	310	53.67 ± 0.81 ^d^
47.02	Luteolin	Flavonoids	360	254.56 ± 3.74 ^a^
56.38	Apigenin	Flavonoids	340	7.32 ± 1.12 ^ef^
57.14	Hesperetin	Flavonoids	310	9.98 ± 1.56 ^ef^
62.40	Baicalein	Flavonoids	340	9.40 ± 0.96 ^ef^

The different letters represent a significant difference, *p* < 0.05.

**Table 4:** Effect of the ethyl acetate fraction of *P. frutescens* leaves on the blood lipid parameters of STZ-induced diabetic rats.

Groups	TC	TG	HDL-C	LDL-C
NC	1.36 ± 0.05 ^c^	0.81 ± 0.06 ^e^	1.16 ± 0.03 ^a^	0.21 ± 0.03 ^c^
HFD	2.40 ± 0.03 ^a^	1.75 ± 0.13 ^a^	0.80 ± 0.07 ^c^	0.55 ± 0.10 ^a^
HFD-ME	1.92 ± 0.11 ^b^	1.52 ± 0.09 ^b^	0.91 ± 0.07 ^bc^	0.38 ± 0.04 ^b^
HFD-HE	1.50 ± 0.08 ^c^	1.05 ± 0.07 ^d^	1.14 ± 0.07 ^a^	0.26 ± 0.03 ^c^
HFD-A	1.76 ± 0.14 ^b^	1.35 ± 0.06 ^c^	1.08 ± 0.14 ^ab^	0.26 ± 0.03 ^b^

TC = total cholesterol; TG = triglyceride; HDL-C = high-density lipoprotein cholesterol; LDL-C = low-density lipoprotein cholesterol; ND = normal diet group; HFD = high-fat diet group; HFD-ME = high-fat diet with medium dose extracts group; HFD-HE = high-fat diet with high dose extracts group; HFD-A = high-fat diet with acarbose group. The different letters represent a significant difference, *p* < 0.05.

## Data Availability

The remaining data are available on request from the corresponding author.
